# New York

**Published:** 1896-08

**Authors:** 


### CONTROL WORK.

*New York.* The New York *Times* in commenting editorially upon the fact that only the cattle of New York county would be examined with tuberculin, while the city's milk-supply came almost wholly from outside, owing to the parsimony of the State legislature, says the work of inspection throughout the State has been suspended in the face of the unanimous recommendations of the State and many local boards of health and a general demand from the public that they be given greater security from the dangers of this source of their food-supply. It highly commends the work done in Massachusetts, and refers to the other States of Connecticut, New Jersey, and Pennsylvania, which are doing like work. Further, that the time is coming when all the dairy-herds in the New England and Middle States will be subjected to the tuberculin-test, for the protection of consumers of dairy-products as well as for the benefit of the owners of cattle. We expressed this opinion some years ago, and it has been confirmed by what has taken place since that time. If State legislatures refuse to provide for the work, the cities will require it to be done throughout their suburban "milk-districts" for the protection of their inhabitants. This State might have been foremost in the movement, but now it lags behind all its neighbors.

